# Distinct cadmium detoxicity response between rice cultivars carrying functional or loss-of-function *OsHMA3* allele

**DOI:** 10.1007/s44154-025-00222-z

**Published:** 2026-10-15

**Authors:** Fuqing Sui, Jing Tian, Ziyuan Yang, Yanzheng Yang, Chang Li, Hongen Liu, Jiali Yan, Peng Zhao

**Affiliations:** 1 Key Laboratory of Soil Pollution Control and Remediation of Henan Province, College of Resources and Environment, Henan Agricultural University, Zhengzhou, China; 2 College of Civil and Architecture Engineering, Chuzhou University, Chuzhou, 239000 China

**Keywords:** Cadmium, *OsHMA3*, Rice varieties, Detoxicity, Tolerance mechanism

## Abstract

**Background and aims:**

Plants encounter various abiotic stress during their growth, making it essential to understand their responses to such challenges. Though function of *OsHMA3* in cadmium (Cd) sequestration into rice root vacuoles has been well characterized, little is known about how different *OsHMA3* alleles influence plant responses to high Cd exposure.

**Methods:**

In this study, we utilized rice cultivars carrying a functional *OsHMA3* allele (9311) and a loss-of-function *OsHMA3* allele (W4) to investigate their differences in Cd detoxification responses. Morphological, physiological and subcellular changes, as well as Cd species were analyzed.

**Results:**

The results indicated that Cd was more extensively transported to shoots in W4 than 9311 as expected. However, W4 exhibited a more pronounced growth inhibition with reductions in shoot and root length, more severe leaf chlorosis with chlorophyll a, b, and carotenoid levels decreased by 31%, 43%, and 45% in W4, respectively, while no significant reductions were observed in 9311. Enhanced cell ultrastructural damage, including membranolytic changes, nuclear membrane lysis, and damage to the nucleus, was noted in W4, while, relatively normal cell structure in 9311. The antioxidant enzyme systems exhibited greater responses in W4 than those in 9311. Additionally, lower levels of nitrate/nitrite, chloride, and aminophenol Cd forms were found in W4 roots, while higher concentrations of these were observed in shoots, indicating distinct Cd detoxicity response between W4 and 9311.

**Conclusion:**

This study underscores the significance of the *OsHMA3* gene in Cd detoxification responses, offering valuable insights into the mechanisms of plant Cd detoxification.

**Graphic Abstract:**

**Figure:**
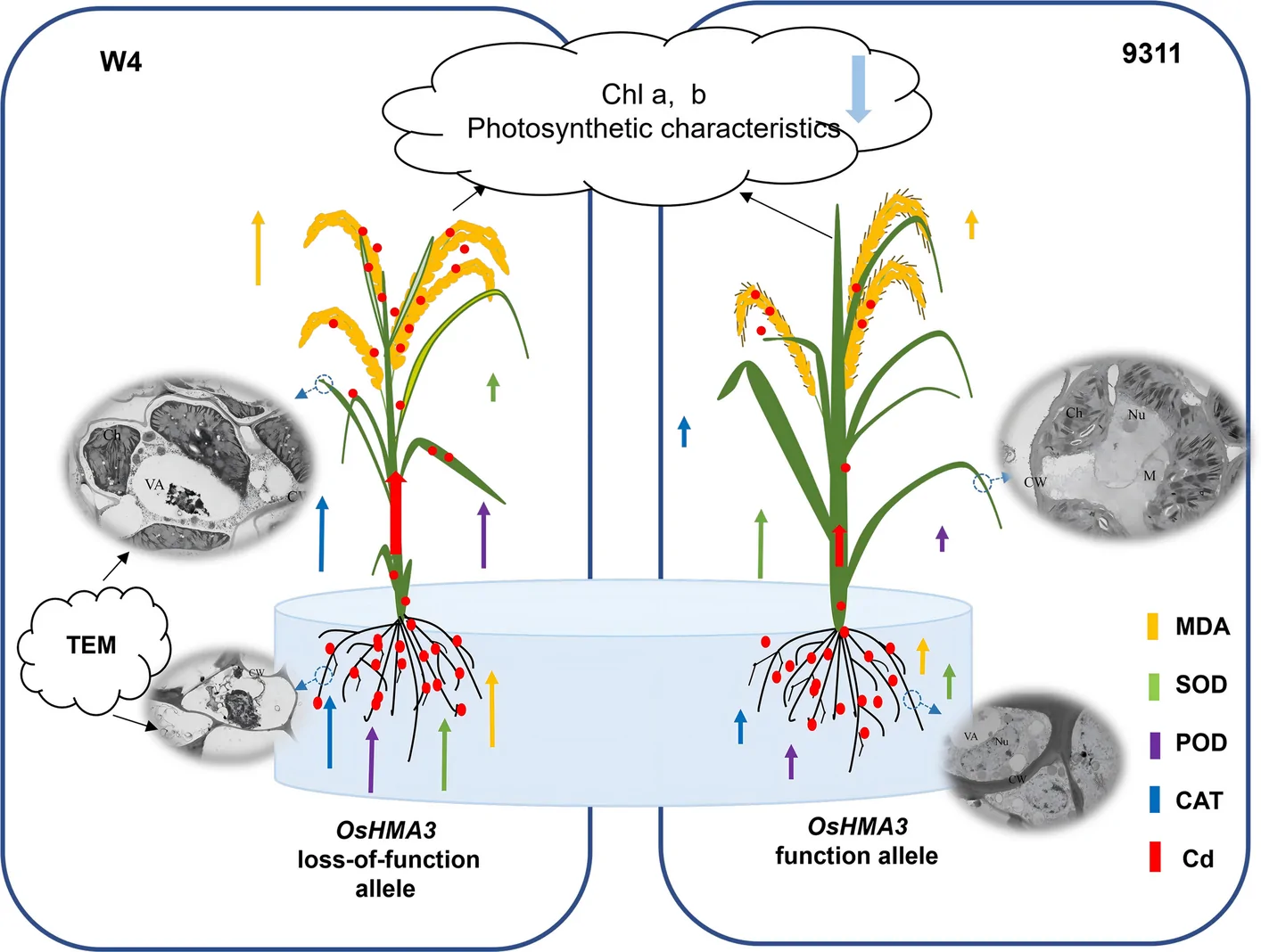

**Supplementary Information:**

The online version contains supplementary material available at https://doi.org/10.1007/s44154-025-00222-z.

## Highlights

1. Rice cultivar with loss-of-function OsHMA3 allele showed greater Cd sensitivity, more severe growth inhibition and leaf chlorosis under Cd toxicity, and leaf chlorosis were induced by Cd toxicity, irrespective of Fe content.

2. More severe Cd toxicity as indicated by oxidative response and cell ultrastructure changes in rice shoot were observed in OsHMA3 function missing cultivar.

3. Significant positive correlation between Cd toxicity and shoot Cd content were observed, which was closely related with OsHMA3 function.

**Supplementary Information:**

The online version contains supplementary material available at https://doi.org/10.1007/s44154-025-00222-z.

## Introduction

As a class I carcinogen, Cadmium (Cd) has been recognized as a public health hazard. Cd has been introduced into the environment due to the rapid industrialization witnessed in recent times (Zhao et al. 2015a). The entry of Cd into agricultural soils primarily occurs through sewage irrigation, improper disposal of industrial and mining waste, and atmospheric deposition (Wang et al. 2019). Cd is highly mobile compared with other elements and easily accumulate in crop edible parts (Clemens et al. 2013). Rice has strong uptake ability for Cd (Chen et al. 2019; Sui et al. 2018), and the frequent reports of Cd-contaminated rice enlarged the risk of Cd entrance into the human body through the food chain (Hu et al. 2016; Rizwan et al. 2016). Many countries and groups has set limits for Cd content in rice, for example, 0.4 mg kg^−1^ limit for the Joint FAO/WHO Expert Committee on Food Additives (JECFA) and Japan, 0.2 mg kg^−1^ limit for China, Korea, Singapore, 0.15 mg kg^−1^ for European Union, 0.10 mg kg^−1^ for Australia and New Zealand (Wang and Zhao 2022). Various strategies have been proposed for Cd contaminated soil remediation (Peera Sheikh Kulsum et al. 2023). For example, biochar (Tian et al. 2023; Zuo et al. 2023), soil amendment (Deng et al. 2020; Li et al. 2019), earthworm (Xiao et al. 2021), microbial agents (Xiao et al. 2021; Zulfiqar et al. 2023), nanomaterials (Ahmed et al. 2023; Jin et al. 2023; Zhao et al. 2021), seed priming technologies (Bano et al. 2024) were used in controlling Cd mobility and reducing Cd accumulation in plant-based food. Despite advancements in preventing and managing heavy metal pollution, Cd minimization in food is still a significant issue (Zhao et al. 2015a). Some of the remediation technologies were conditional (Zuo et al. 2023). Therefore, it is imperative to implement comprehensive measures to control heavy metal pollution in order to safeguard environmental integrity as well as human health (Chen et al. 2018; Wang et al. 2019).

Cadmium is not essential and is toxic for plants. In rice, the uptake and translocation of Cd are facilitated by a variety of transporters (Clemens and Ma 2016; Zhao et al. 2022). The root, being the first barrier for Cd uptake, serves as the main organ for Cd entrance into plant (Clemens et al. 2013). *OsNramp*, *OsIRT* and *OsZIP* family transporters are involved in rice root Cd uptake (Chang et al. 2020; Ishikawa et al. 2012; Jiang et al. 2021; Sasaki et al. 2012; Sui et al. 2018; Tan et al. 2020; Tang et al. 2017; Yang et al. 2014). While *OsLCT* and *OsCCX* are involved in Cd loading into rice grains (Hao et al. 2018; Uraguchi et al. 2011). *OsHMAs* play important roles in Cd translocation from root to shoot (Takahashi et al. 2012; Ueno et al. 2010). In rice, *OsHMA3* was firstly cloned by map-based cloning technology using a population derived from a high Cd cultivar Anjana Dhan and a low Cd cultivar Nipponbare (Ueno et al. 2010). Further function analysis showed that *OsHMA3* mainly was expressed on tonoplast of rice root cells, playing a crucial role in transporting Cd into vacuoles for sequestration, and thus limiting Cd transportation to above-ground tissues (Ueno et al. 2010). The cloning of *OsHMA3* expressed previously reported quantitative trait loci for high grain Cd accumulation on chromosome 7 (Ishikawa et al. 2010; Ueno et al. 2009). Overexpression of *OsHMA3* results in greater Cd sequestration in root cell vacuoles and reducing Cd transport to shoots and grains in both Indica and Japonica rice cultivar (Lu et al. 2019; Sasaki et al. 2014), and even wheat (Zhang et al. 2020). Similarly, *OsHMA3* expression level changes caused by the promoter region changed Cd accumulation in rice grains (Liu et al. 2019). Overexpression of *OsHMA3* also alleviate Cd-induced lateral root emergence and root growth by *OsPIN*-mediated auxin distribution (Wang et al. 2020). Conversely, amino acid substitution at 80th (Ueno et al. 2010), 380th (Yan et al. 2016), and 512th (Sun et al. 2019) position, 153 bp deletion in exon 7 (Miyadate et al. 2011), and 42 bp deletion in the ATP-binding domain (Sui et al. 2019) would disturb function of *OsHMA3* and lead to a higher translocation ratio of Cd from roots to shoots and Cd accumulation in rice grains. By the way, the 42 bp deletion in the ATP-binding domain seem to cause a completely loss-of-function of *OsHMA3* compared with other alleles (Sui et al. 2019). Further analysis of showed that *HMA3* in Arabidopsis (Chao et al. 2012; Gravot et al. 2004; Morel et al. 2009), wheat (Maccaferri et al. 2019; Zhang et al. 2020), barely (Lei et al. 2020), soybean (Wang et al. 2018, 2012), maize(Cao et al. 2019; Chen et al. 2023; Tang et al. 2021), Zn/Cd hyperaccumulator Sedum plumbizincicola (Liu et al. 2017) and cabbage (Zhang et al. 2019) also play similar roles in Cd uptake and translocation.

Though function of *OsHMA3* has been well characterized and abundant alleles with natural variations were found, little is known on how rice cultivars carrying different alleles of *OsHMA3* response to high Cd exposure. Here, using rice cultivars W4 and 9311, which carries loss-of-function functioning or functional *OsHMA3* allele (Liu et al. 2019; Sui et al. 2019), we investigate the role of root vacuole Cd sequestration by *OsHMA3* in Cd detoxification. The results showed that rice cultivar with a loss-of-function *OsHMA3* allele exhibited increased Cd sensitivity, more pronounced growth inhibition, and greater Cd antioxidant enzyme system response. Additionally, oxidative response and changes in cell ultrastructure were more severe in *OsHMA3* loss-of-function cultivar. These findings suggest that root vacuole Cd sequestration plays a crucial role in Cd detoxification and tolerance.

## Results

### Rice seedlings growth response under Cd stress

**Fig. 1:**
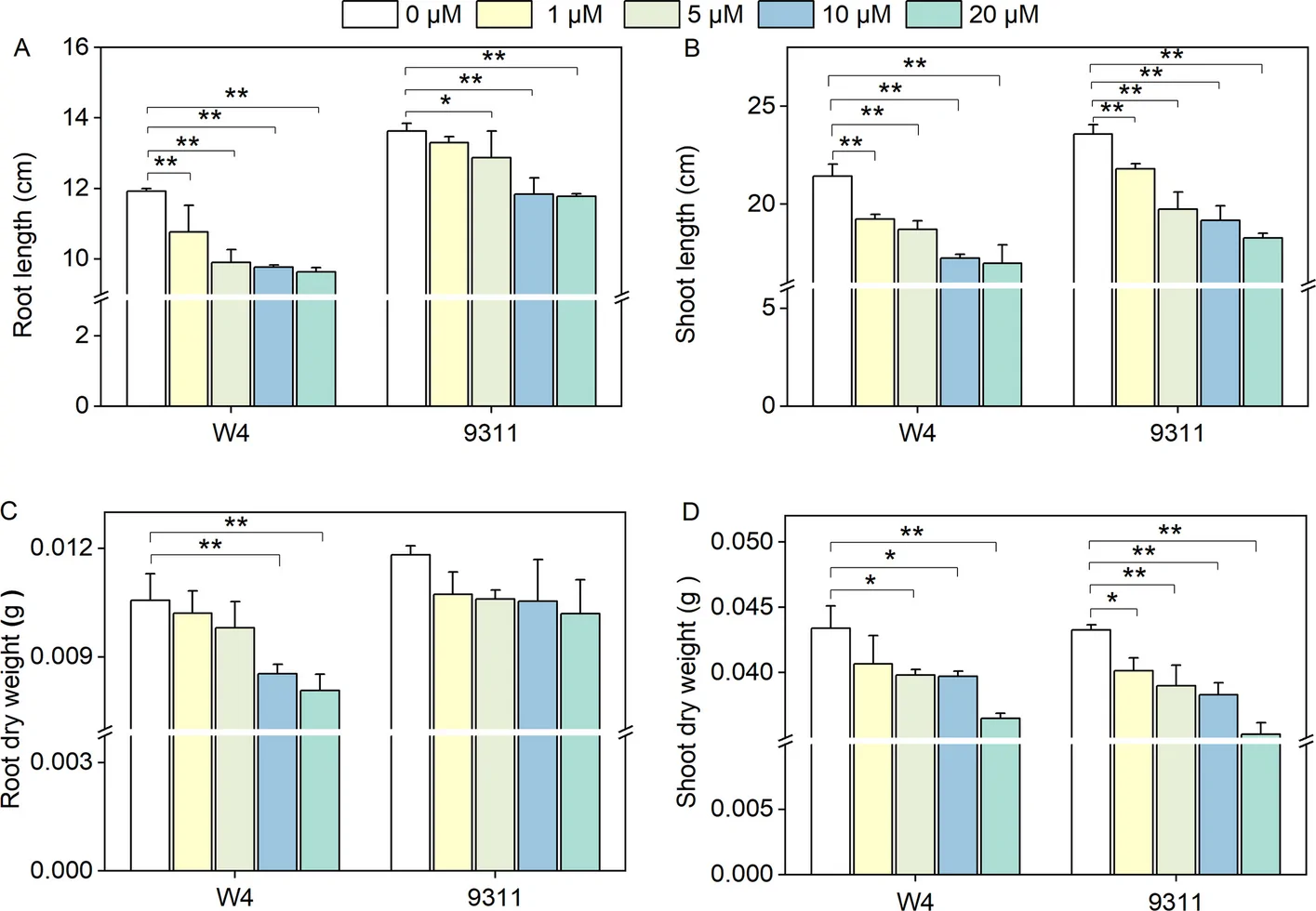
Growth of W4 and 9311 under Cd concentration gradient. The seedlings were hydroponically exposed to 0, 1, 5, and 10 μM Cd for 14 d. **A** Root length. **B** Shoot height. **C** Root dry weight. **D** Shoot dry. Means ± S.D., Tukey's test. *, *P* < 0.05; **, *P* < 0.01, *n* = 3

A concentration gradient was established to evaluate the physiological changes in rice cultivars W4 and 9311 under Cd stress. Both cultivars displayed significant growth inhibition under high Cd exposure (10 and 20 μM Cd), with W4 experiencing more severe growth suppression than 9311 (Fig. 1). Root length in W4 decreased by 10%-23% compared to the control, while in 9311, it decreased by 2%-14% under 1–20 μM Cd treatment, respectively (Fig. 1A). Similarly, shoot length in both cultivars was notably reduced, with W4 showing a 12%, 5%, 10%, 7% greater reduction than 9311 (Fig. 1B). W4 root biomass was significantly decreased by 19% and 24% under high Cd treatment, while 9311 root biomass remained unaffected (Fig. 1C). However, the reduction in shoot biomass in both W4 and 9311 under high Cd treatment was comparable, with decreases of 8%, 16% and 11%, 18%, respectively (Fig. 1C, D).

**Fig. 2:**
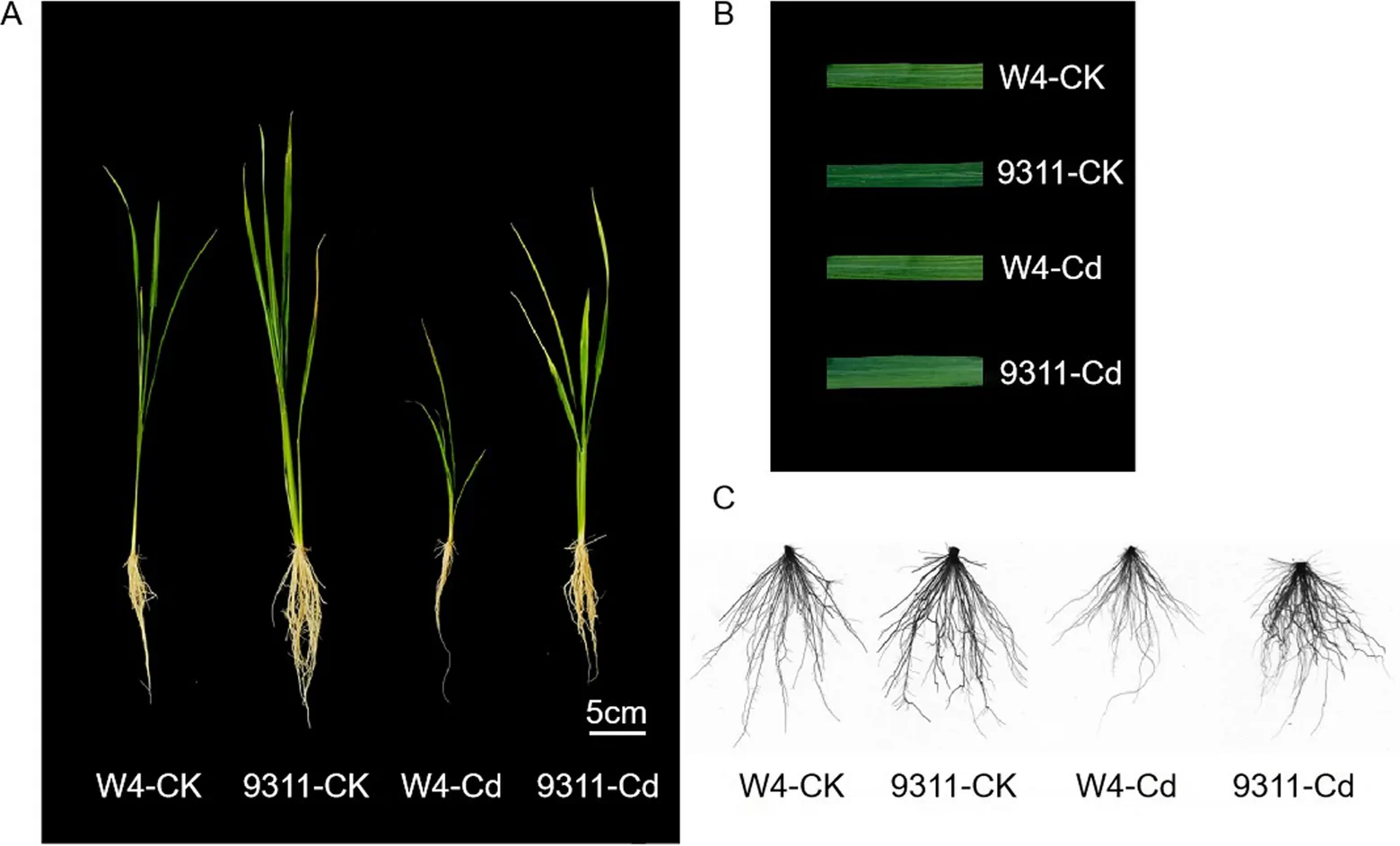
Morphological changes in rice seedlings under high cadmium exposure were observed. **A** The overall growth phenotype of rice seedlings was examined. **B** Rice leaf chlorosis was observed, with representative leaves sampled from the same seedlings as in (**A**). **C** Rice root morphology was observed using a root scanner. Rice seedlings of varieties W4 and 9311 were exposed to a 10 μM cadmium treatment for 14 days

The toxicity of W4 and 9311 seedlings was further characterized by exposure to 10 μM Cd, a concentration known to cause growth arrest. W4 showed higher sensitivity to Cd compared to 9311, with a notable decrease in seedling height and increased leaf chlorosis observed (Fig. 2A, B). Chlorophyll a, b, and carotenoid content in W4 decreased by 31%, 43%, and 45% respectively, while no significant reduction was observed in 9311 (Table S1). W4 roots exhibited increased sensitivity to Cd stress as shown by a root scanner (Fig. 2C). In W4, root length, surface area, and tip number were significantly reduced by 33%, 43%, and 28% respectively, while no notable changes were observed in 9311 (Table S2). Both cultivars experienced a significant reduction in root volume, while root diameter remained unaffected (Table S2). To further understand the physiological changes in rice seedlings under Cd stress, photosynthetic parameters were examined (Table S3). The *T*_*r*_ of W4 and 9311 decreased by 72% and 54%, respectively. W4 exhibited significant reductions in *G*_*s*_ by 68% and Ci by 24%. *P*_*n*_ was not impacted by Cd stress (Table S3). In summary, these findings suggest that W4 displays greater sensitivity to Cd toxicity compared to 9311.

### Cell ultrastructure changes upon Cd stress

**Fig. 3:**
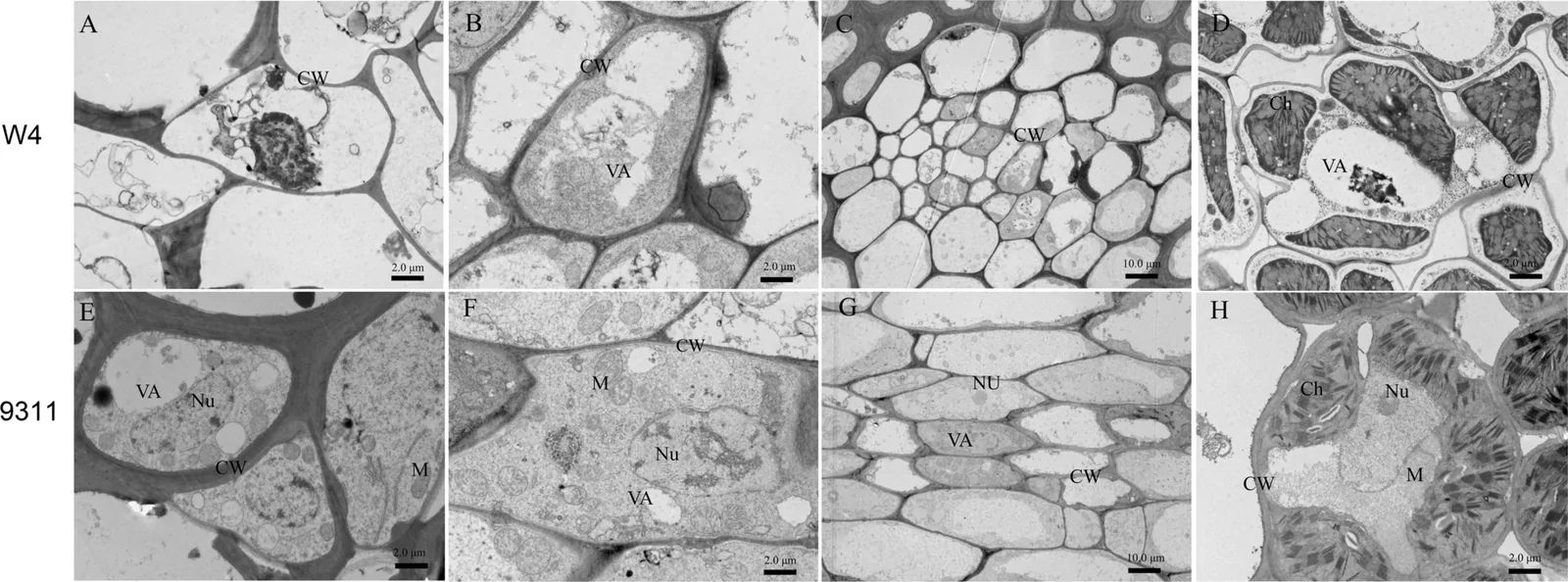
Transmission electron microscope (TEM) observation of ultrastructural cell changes upon Cd stress. The rice seedlings were hydroponically treated with 10 μM CdCl_2_ for 14 d. Representative images were displayed from independent 3 replicates. **A**, **E** Cross section of root. (**B**, **F**) Basal stem. **D**, **H** Leaf blade. **C**, **G** Enlarged field image of (B, F). Nu, nucleus; Ch, chloroplast; M, mitochondria; CW, cell wall; VA, vacuole

Distinct cellular alterations were observed in both W4 and 9311 under 10 μM Cd treatment (Fig. 3). In W4, membranolytic effects such as nucleus membrane lysis, damaged nucleus, deformed cell wall towards the inside, and thinner cell wall were observed (Fig. 3A). Conversely, 9311 showed reduced cell wall deformation and nucleus breakdown, intact subcellular organelles, and well-developed fluid bubbles (Fig. 3E). Additionally, the basal stem of W4 exhibited an unstable cell wall, obscured nucleus and nucleolus (Fig. 3B, C), while 9311 basal stems displayed transparent cell walls free of precipitation and intact cell structure mitochondria (Fig. 3F, G). Furthermore, W4 leaves showed swelled cell walls, blurred or invisible vacuoles and nuclei, as well as deformed, swollen, rounded, fingerprint-like, and vacuolated chloroplasts (Fig. 3D). In contrast, 9311 leaves exhibited a clean inner cell wall without precipitation, a complete nucleus, neatly arranged chloroplasts close to the cell wall, and an intact mitochondrial structure (Fig. 3H).

### Cd and other metals uptake and translocation

**Fig. 4:**
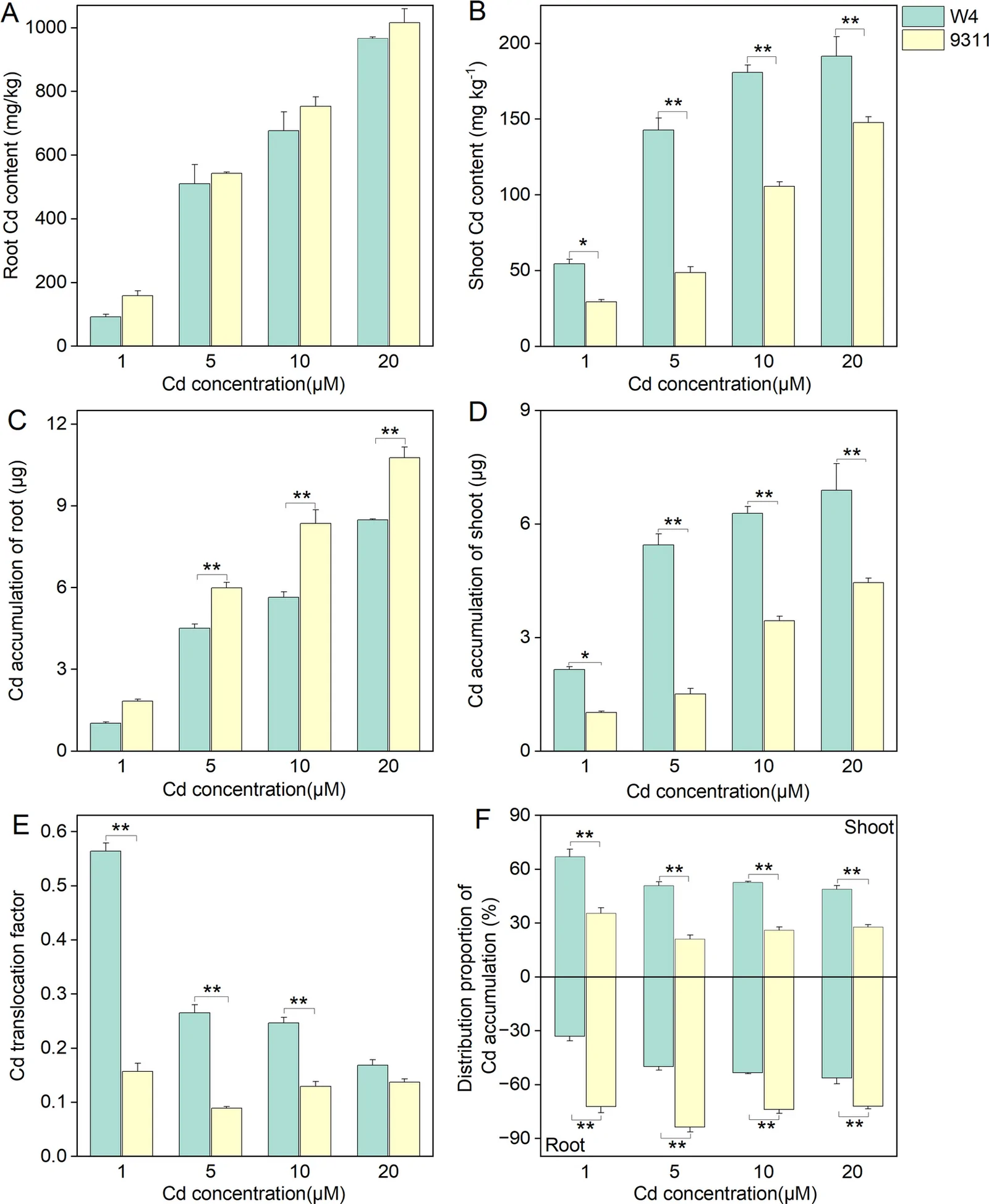
Cd uptake and translocation of W4 and 9311 under different Cd treatments. **A** Root Cd content. **B** Shoot Cd content. **C** Root Cd accumulation. **D** Shoot Cd accumulation. **E** translocation factor. **F** Cd distribution ratios. W4 and 9311 seedlings were treated at 0, 1, 5, 10, and 20 μM Cd for 14 days. Data were shown as means ± S.D., Tukey's test, *, *P* < 0.05; **, *P* < 0.01, *n* = 3

Cd uptake and translocation in response to Cd exposure were compared between cultivars W4 and 9311 (Fig. 4). Although both cultivars had similar Cd content in their roots, the shoot of W4 had significantly higher Cd content compared to 9311, with increases of 47%, 66%, 42%, and 18% under 1, 5, 10, and 20 μM Cd treatments, respectively (Fig. 4A, B). Root Cd accumulation in W4 was notably lower than that in 9311, with reductions of 77%, 34%, 48%, and 26% under Cd treatments (Fig. 4C). Conversely, shoot Cd accumulation in W4 was consistently higher than in 9311, with increases of 54%, 72%, 46%, and 35% under all Cd treatments, respectively (Fig. 4D). These findings support previous reports that *OsHMA3* plays a role in transporting Cd from the cytoplasm into vacuoles for sequestration in rice root cells, resulting in reduced Cd translocation to above-ground tissues in 9311, while more Cd is transported to the shoot in W4 (Liu et al. 2019; Sui et al. 2019). Additionally, Fe, Mn, Cu, and Zn content in the roots and shoots of both rice cultivars were analyzed. No differences in Fe content were observed between the roots and shoots of the two cultivars (Fig. S1). However, lower Mn, Cu, and Zn content was detected in the shoot of W4 compared to 9311, while Cu and Zn content in the root of W4 were higher than in 9311 (Fig. S1).

### Antioxidant system enzymes response upon Cd stress

**Fig. 5:**
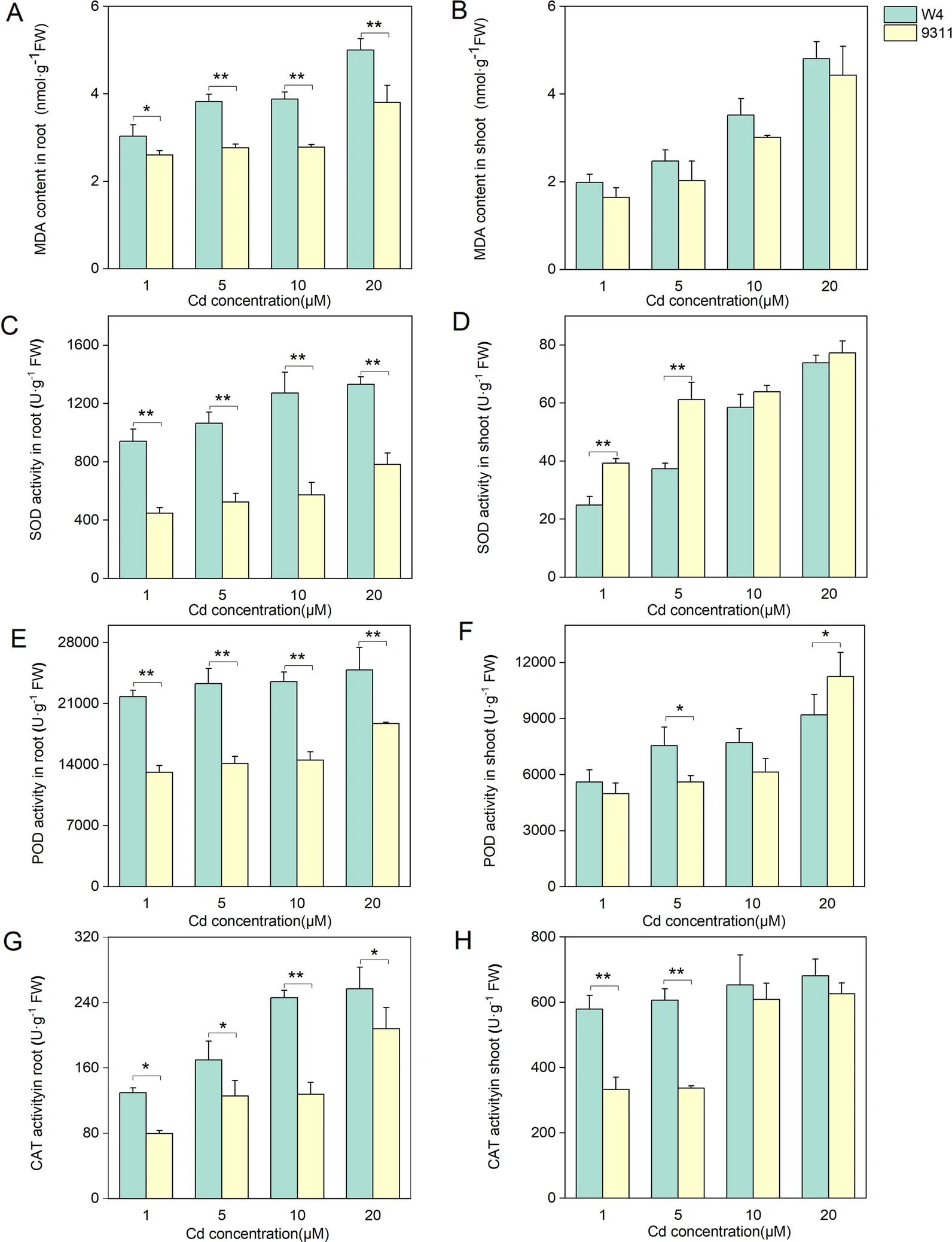
Effects of Cd stress on MDA contents and antioxidant enzyme activities. **A** Root MDA content. **B** Shoot MDA content. **C** Root SOD activity. **D** Shoot SOD activity. **E** Root POD activity. **F** Shoot SOD activity. **G** Root CAT activity. **H** Shoot CAT activity. Data were shown as means ± S.D., *n* = 3. Tukey’s test, *, *P* < 0.05; **, *P* < 0.01

To analyze the physiological responses to Cd toxicity in W4 and 9311, we measured the MDA content and antioxidant enzyme activities, and the results showed contrasting responses (Fig. 5). Specifically, the MDA content in the roots and shoots of rice seedlings increased significantly with higher Cd concentrations. W4 roots had a higher MDA content compared to 9311 by 17%-39%, while no significant difference was observed in the shoots of both cultivars (Fig. 5A, B). The SOD activities in W4 roots were notably higher than in 9311, ranging from 70 to 122% under different Cd treatments (Fig. 5C). However, at sub-toxic concentrations of 1 μM and 5 μM Cd, SOD activity in W4 shoots was lower by 37% and 39% compared to 9311 (Fig. 5D). Similarly, the POD activities in W4 roots were higher than in 9311 by 33% to 66%, with a slight decrease in W4 shoot under 20 μM Cd treatment (Fig. 5E, F). Additionally, CAT activity in W4 roots was consistently higher than in 9311, ranging from 24 to 93% under different Cd treatments (Fig. 5G, H). These findings suggest that the isolation of Cd into the vacuole by *OsHMA3* in roots plays a crucial role in Cd detoxification.

### Cd subcellular distribution and Cd chemical forms in plants

**Fig. 6:**
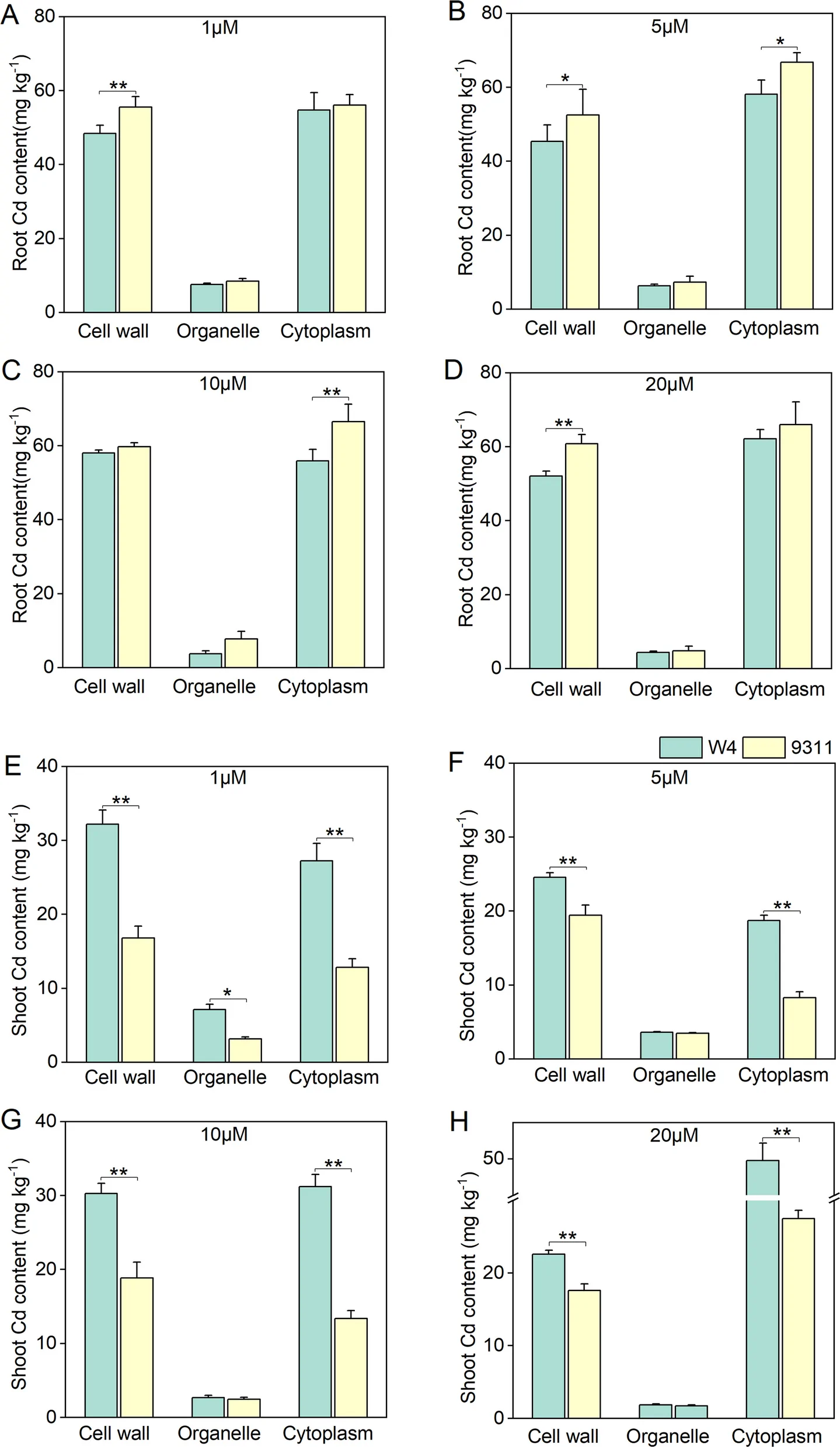
Effects of exogenous Cd on subcellular distribution of Cd in seedlings of different rice varieties. Cd content in root part of (**A**) 1 μM treatment, (**B**) 5 μM treatment, (**C**) 10 μΜ treatment and (**D**) 20 μM treatment. Cd content in shoot part of (**E**) 1 μM treatment, (**F**) 5 μM treatment, (**G**) 10 μΜ treatment and (H) 20 μM treatment. Data were means ± S.D., *n* = 3. Tukey’s test, *, *P* < 0.05; **, *P* < 0.01

The subcellular components Cd content were fractioned to investigate the Cd detoxication mechanisms of W4 and 9311. There was no significant difference in organelle fraction Cd concentration between W4 and 9311 under all Cd concentration treatments. In rice roots, cell wall fraction and cytoplasm fraction Cd content in W4 were slightly lower than that in 9311 (Fig. 6A-D). However, a remarkable difference was observed in the shoots of W4 and 9311. The cell wall fraction Cd content of W4 shoot was 26%-91% higher than that of 9311, while the cytoplasm Cd content of W4 shoot was 81%-133% higher than that of 9311, under the tested Cd treatment (Fig. 6E-H).

**Fig. 7:**
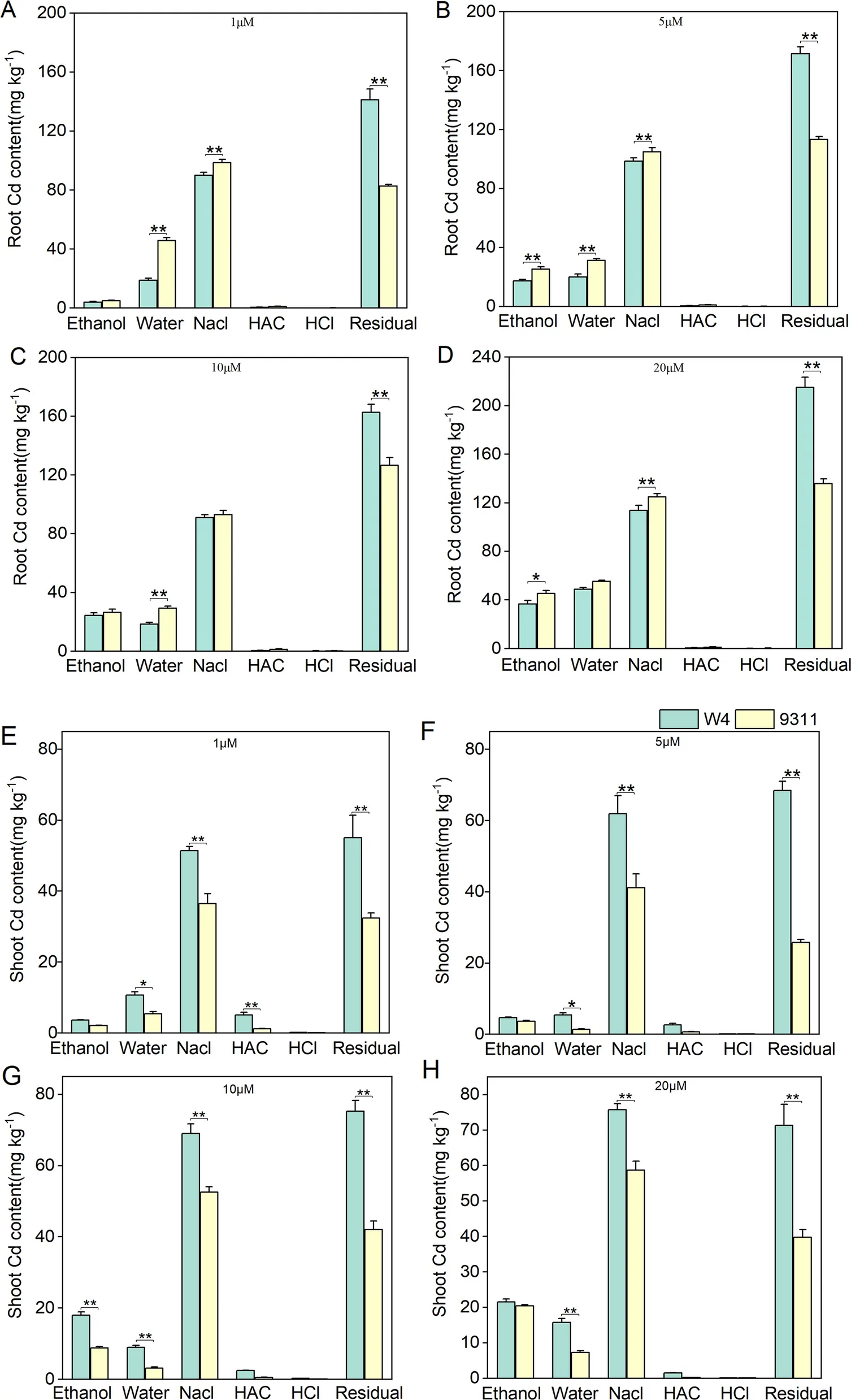
Cd speciation differences in W4 and 9311 under Cd stress. Cd content in root part of (**A**) 1 μM treatment, (**B**) 5 μM treatment, (**C**) 10 μΜ treatment and (**D**) 20 μM treatment. Cd content in shoot part of (**E**) 1 μM treatment, (**F**) 5 μM treatment, (**G**) 10 μΜ treatment and (H) 20 μM treatment. Data were shown as means ± S.D., *n* = 3. Tukey’s test, *, *P* < 0.05; **, *P* < 0.01

Based on these results, Cd species were further fractionated (Fig. 7). The Cd content extracted by 80% ethanol in W4 was 8%-31% lower than that in 9311, the water-soluble Cd content in W4 was 12%-59% lower than that in 9311, under different Cd treatments, respectively. On the other hand, NaCl extractable Cd and residual Cd fractions in W4 was 2%-10%, 28%-71% higher than that in 9311 under 1–20 μM Cd treatments, respectively. No significant difference in Cd content extracted by HAC and HCl between W4 and 9311 was observed (Fig. 7A-D). However, a greater consistency of Cd speciation changes was observed in the shoots of both cultivars. Under different Cd treatments, Cd extracted by ethanol, water, NaCl, HAC, HCl, and residual fractions in W4 were 5%-106%, and 97%-290%, 29%-50%, 72%-86%, 21%-159%, and 70%-166% higher than that in 9311, respectively (Fig. 7E-H).

### Principal Component Analysis (PCA) of physiological indexes and Cd content

**Fig. 8:**
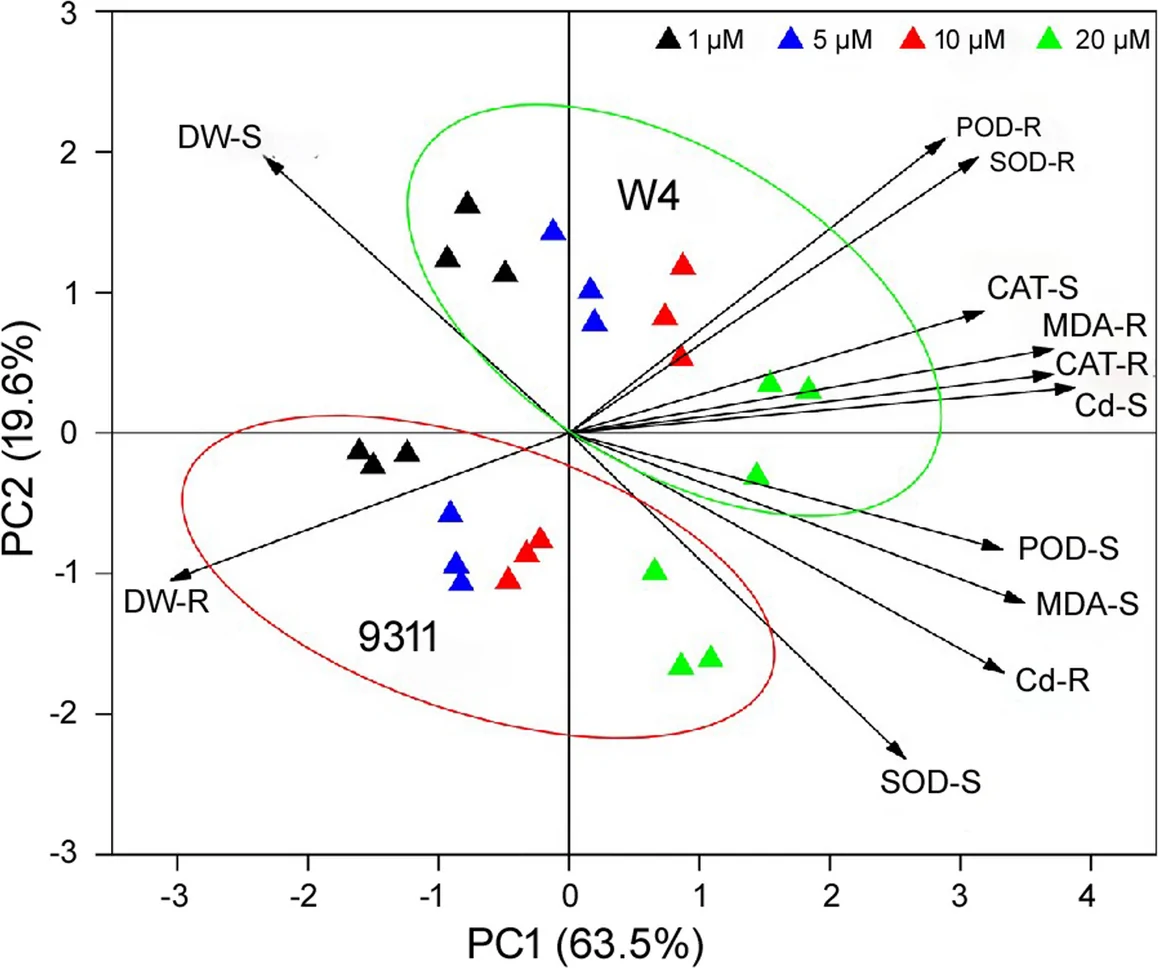
Principal component analysis was conducted on two rice varieties (W4 and 9311) exposed to different concentrations of Cd (1, 5, 10, or 20 μM) to assess physiological indicators, Cd content, and antioxidant enzyme activity. The physiological indicators included DW-S (shoot dry weight), DW-R (root dry weight), Cd-S (shoot Cd content), Cd-R (root Cd content), MDA-R (root MDA content), SOD-R (root SOD activity), CAT-R (root CAT activity), POD-R (root POD activity), MDA-S (shoot MDA content), SOD-S (shoot SOD activity), CAT-S (shoot CAT activity), and POD-S (shoot POD activity)

PCA was utilized to analyze the physiological response to Cd stress. The cumulative contribution ratio of the PCA was 83.1%, with PC1 contributing the most at 63.5% and PC2 contributing 19.6% (Fig. 8). The distribution of rice seedlings under 1 μM treatment was similar, but the physiological response of W4 differed significantly from that of 9311 under high Cd treatment. The oxidative response and mineral elements concentration were mainly in the first quadrant as Cd levels increased, with W4 showing a stronger correlation and more intense response compared to 9311. The notable difference between W4 and 9311 under high Cd treatments (5, 10, and 20 μM) suggests that Cd stress induced more physiological changes in W4, possibly due to the role of *OsHMA3*.

## Discussion

Rice, as a staple food, is at risk of heavy metal toxicity in polluted environments. Understanding the mechanisms by which rice defends against Cd stress is crucial for sustainable food production and human health protection. This study observed the response of rice cultivars with different *OsHMA3* alleles to Cd stress and analyzed potential mechanisms. By studying these cultivars, we elucidated the impact of the absence of vacuole isolation transportation on Cd detoxification.

### Cd toxicity causes more severe growth inhibition in cultivars carrying the OsHMA3 loss-of-function allele

Contrasting growth differences were observed between cultivars carrying the functional *OsHMA3* allele and those that did not. Both cultivars exhibited significant growth arrest when exposed to high levels of Cd, resulting in a reduction in shoot and root length as well as biomass. This is consistent with previous studies which have shown that Cd can significantly reduce shoot and root lengths, inhibit plant growth, and decrease biomass in wheat plants (Wu et al. 2005; Zhu et al. 2022). Notably, a greater growth inhibition was observed in W4, which carries a loss-of-function *OsHMA3* allele. W4 exhibited a more pronounced reduction upon Cd toxicity in shoot and root length as well as biomass compared to cultivar 9311 (Figs. 1 and 2). Additionally, there was a rapid decline in photosynthetic content as Cd levels increased, serving as a key indicator of plant stress (Qin et al. 2018). W4 also displayed more severe leaf chlorosis and decreased chlorophyll content (Fig. 2B, Table S1). The greater reduction in plant height, root weight, and shoot chlorophyll content under Cd stress in W4 can be attributed to the function of *OsHMA3*. Previous studies have shown that Cd disrupts *OsPIN*-mediated auxin distribution, leading to the inhibition of lateral root emergence (Wang et al. 2020). When examining root parameters in both rice seedlings exposed to high Cd levels, it was found that the root surface area and tip number were significantly reduced in W4, while no significant changes were observed in 9311 (Table S2). These findings suggest that rice seedlings experience greater Cd toxicity in the absence of Cd sequestration into root cells, highlighting the important role of root vacuoles in protecting plants from Cd toxicity (Wang et al. 2020).

It has been previously confirmed that Cd accumulation impairs various metabolic processes, such as nutrient absorption and photosynthesis, thus strongly inhibited plant growth and reduced biomass (Zhu et al. 2022). Numerous studies have also reported variations in the transport and accumulation of Cd in cereal crops (Ma et al. 2021). In the present study, mineral elements and Cd levels were measured in two rice cultivars to assess the roots nutrient uptake status under Cd stress. W4 exhibited higher Cd levels in the shoot and higher Cd translocation ratio than that of 9311, despite having similar Cd levels in the root (Fig. 4). A decline in Mn, Cu, and Zn contents, along with comparable Fe contents, was observed in the shoot of W4, while higher Cu and Zn levels were noted in the root of W4 compared to 9311 (Fig. S1). These findings indicate that the leaf chlorosis induced by Cd was not caused by Fe deficiency, contrary to previous reports linking Cd stress to Fe-deficient leaf chlorosis (Yoshihara et al. 2006). The more pronounced leaf chlorosis in W4 may be attributed to the transport of mineral elements to the above-ground tissues induced by elevated Cd exposure.

### More severe ultrastructure changes upon Cd stress were observed in the OsHMA3 loss-of-function allele cultivar

Previous studies have extensively documented the ultrastructural changes in plant cells under Cd stress. High Cd stress leads to chloroplast deformation, reduction in nucleus size, and changes in cell membrane structure (Gu et al. 2023; Wang et al. 2022). Cd exposure results in alterations to cellular morphology, with black precipitates appearing on both sides of the cell wall and vacuoles, thickening and deformation of the cell wall, and damage to organelles (Gu et al. 2023). Bundle sheath cell chloroplasts are particularly affected by Cd (Vaculík et al. 2015). In this study, significant damage to the root cell structure of W4 due to Cd stress was observed, while the cell wall of 9311 remained intact (Fig. 3). Reduced cell wall deformation and nucleus breakdown, intact subcellular organelles and well development of fluid bubbles were observed in 9311 in both root and basal stem region and leaf (Fig. 3). Above-ground tissues of W4 exhibited more severe cell damage, characterized by cell wall deformation, chloroplast deformation, and thinning of the chloroplast matrix, which was positively correlated with Cd content. This increased damage in W4 may be attributed to the dysfunction of *OsHMA3*, leading to higher Cd transport to above-ground tissues.

### Cd detoxicity difference between rice cultivars carrying functional or loss-of-function OsHMA3 allele

A dynamic equilibrium exists between the synthesis and elimination of free radicals in plant cells. An excess of Cd in plants not only hinders plant development but also disrupts cellular redox balance, leading to the generation of ROS (Chen et al. 2021; Rui et al. 2018). The activity of antioxidant enzymes such as POD, APX, and CAT is heightened in response to Cd-induced oxidative stress in plants, with the corresponding genes also being upregulated (Fornazier et al. 2002). MDA is a byproduct of membrane lipid peroxidation, serves as a crucial biomarker for assessing peroxidation levels. Previous studies have shown that elevated Cd exposure results in the overproduction of ROS, as well as increased MDA and H_2_O_2_ content in wheat (Guo et al. 2019). The activities of POD, SOD, and CAT serve as indicators of the severity of Cd stress on plants (Din et al. 2023). In the current investigation, Cd stress notably increased the root MDA content in both rice cultivars, with a more pronounced increase observed in W4 compared to 9311 (Fig. 5). Despite this, SOD activity in the roots remained stable and even increased, ensuring the effective scavenging of free radicals. Furthermore, the higher CAT activity in W4 compared to 9311 suggests a stronger oxidative stress response. Overall, in the absence of *OsHMA3* function, rice seedlings exhibit a heightened oxidative response to Cd toxicity. The isolation and transport of Cd into the vacuole play a crucial role in Cd detoxification.

### Cd subcellular fraction and chemical binding forms

Cd spreads across various organelles once it enters plant cells through the roots. The root cell wall acts as the primary barrier against heavy metal entry and is essential for heavy metal sequestration. Previous synchrotron-based techniques revealed that 66%–92% of the Cd in rice grain was present as Cd thiolate complexes likely in the form of Cd bound with thiol-rich proteins (Gu et al. 2020). In this study, the binding of Cd to the W4 root cell wall was notably lower compared to thah in 9311. However, a significantly higher Cd content was found in the cell wall and cytoplasm fraction of W4 shoot (Fig. 6). These findings indicate that the absence of *OsHMA3* function in Cd detoxification in roots results in a higher distribution of Cd to the cytoplasm in the above-ground tissues, leading to more severe toxicity. This aligns with previous research showing that the soluble fraction immobilizes and accumulates Cd, restricting its movement during detoxification (Wei et al. 2023). The results strongly suggest that the cell wall and cytoplasm play a crucial role in combating Cd toxicity, and the function of *OsHMA3* is closely linked to the ability to respond to Cd stress in the W4 and 9311.

There are significant differences in the transport capacity of different chemical forms of Cd in plants, which impact Cd transport and toxicity. To further elucidate the role of *OsHMA3* in Cd detoxification, Cd species in plants were fractionated. Specifically, ethanol was used to extract nitrate/nitrite, chloride, and aminophenol-bound Cd, water for water-soluble Cd of organic acid, NaCl for pectates and protein-bound Cd, and HAc for undissolved Cd phosphate and residual forms (Wu et al. 2005). Lower Cd content was extracted by ethanol, water, and NaCl, with higher residual Cd content in the W4 root. Conversely, significantly higher Cd content extracted by ethanol, water, and NaCl was observed in the shoot of W4 compared to 9311 (Fig. 7). These findings suggest that in the absence of OsHMA3 function, more Cd is transported to above-ground tissues and Cd in W4 is more efficiently transferred than in 9311, underscoring the importance of *OsHMA3* in Cd detoxification. Additionally, the residual form of Cd was found to be increased in the shoot of W4 compared to 9311. In apple rootstocks, strategies such as immobilizing Cd in the cell wall and converting it into pectate- or protein-bound forms, as well as undissolved Cd phosphate forms, may serve as primary mechanisms to reduce Cd mobility and toxicity (Zhou et al. 2017). To further explore Cd detoxification in the presence or absence of *OsHMA3* function, PCA of Cd content and physiological indexes was conducted. The results revealed that physiological responses were more pronounced under toxic Cd levels, showing a stronger correlation in the absence of *OsHMA3* function, albeit similar in low Cd treatment. This underscores the significance of root vacuole Cd isolation transport mediated by *OsHMA3* in Cd detoxification.

Taken together, loss-of-function of *OsHMA3* lead to more severe Cd toxicity response compared with that rice cultivars carrying functional alleles, the present investigation underscores the crucial role of *OsHMA3* in Cd detoxication.

## Conclusion

Rice cultivars carrying the loss-of-function *OsHMA3* allele showed greater sensitivity compared to those with the functional *OsHMA3* allele when exposed to the same Cd treatment. The absence of *OsHMA3* function resulted in increased damage at the ultrastructural cell level, higher levels of MDA content, and a more pronounced response from antioxidant enzyme systems. Elevated levels of Cd were observed in both the cell wall fraction and cytoplasm fraction, as well as increased mobility of Cd in the shoot. Overall, these findings suggest that *OsHMA3* plays a crucial role in limiting the translocation and detoxification of Cd.

## Material and methods

### Plant culture

Rice cultivars W4 and 9311 were obtained in the present study, both belongs to the indica subgroups. W4 was provide by professor Guiquan Zhang from South China Agricultural University (Teng et al. 2011), and 9311 was acquired from professor Qianqian from China National Rice Research Institute (Liu et al. 2019), agricultural traits and Cd accumulation was well characterized in the previous studies (Liu et al. 2019; Sui et al. 2019). Rice seeds W4 and 9311 were disinfected with 3% hypochlorous acid for 30 min and then rinsed thoroughly with deionized water. The sterilized seeds germinated at 37 °C for 3 days before being sowed on a floating net in a 0.5 mM CaCl_2_ solution. The uniform seedlings were then transferred for hydroponic culture in half-strength Kimura B nutrient solution (pH 5.6), which was renewed every 3 days (Ma et al. 2001). The plants were grown in a natural light greenhouse with day/night temperatures of 37 °C/25 °C. Rice seedlings of W4 and 9311, pre-cultured for one month, were then transferred for CdCl_2_ treatment (0, 1, 5, 10, and 20 μM) for 14 days in a 5 L plastic bucket. Each treatment was replicated 3 times and the solution was renewed every 3 days. Growth parameters were recorded and the seedlings were used for analysis.

### Sampling and determination

Rice roots were soaked in 2 mM EDTA-Na_2_ for 5 min to remove surface adsorbed Cd, then rinsed three times with ultrapure water, blotted dry, and finally dried at 65 °C for 3 days. The dry weight samples were recorded and ground. A 0.2 g sample was digested using 5 mL of mixed acid (HNO_3_/HClO_4_, 87/13) in a heating block (ED54, LabTECH, Boston, USA) (Zhao et al. 1994). Cd concentration was analyzed using Inductively Coupled Plasma Mass Spectrometry (ICP-MS, ICAP RQ, Thermo Fisher Scientific Co. Ltd., Waltham, USA). Standard sample rice flour 1568b was utilized for quality control, with a recovery ratio ranging from 93 to 105%, indicating the reliability of the sample analysis. Cd accumulation, translocation factor and distribution proportion were calculated as follows:

$$\mathrm{Cd}\;\mathrm{accumulation}\;\mathrm{of}\;\mathrm{root}\;\left(\mu\mathrm{g}\right)=\mathrm{Root}\;\mathrm{Cd}\;\mathrm{content}\times\mathrm{Root}\;\mathrm{biomass}$$

$$\mathrm{Cd}\;\mathrm{accumulation}\;\mathrm{of}\;\mathrm{shoot}\;\left(\mu\mathrm{g}\right)=\mathrm{Shoot}\;\mathrm{Cd}\;\mathrm{content}\times\mathrm{Shoot}\;\mathrm{biomass}$$

$$\text{Cd translocation factor}=\frac{\text{Shoot Cd content}}{\text{Root Cd content}}$$

$$\text{Distribution proportion of Cd accumulation }(\%)=\frac{\text{Cd accumulation of shoot}}{\text{Cd accumulation of shoot}+\text{ Cd accumulation of root}}$$

### Root morphology and photosynthetic parameter observation

Root morphological parameters, including total root length, total root surface area, average diameter, total root volume, and total root tip number, were measured using a root canister as previously reported (WinRHIZO 2009, Canada) (Nie et al. 2017). Photosynthetic parameters, including net photosynthetic rate (*P*_*n*_), stomatal conductance (*G*_*s*_), and transpiration rate (*T*_*r*_), were assessed using a portable photosynthetic analyzer (LI-6400P, Li-Cor, USA) prior to harvest. Chlorophyll was extracted from 0.20 g fresh leaves by cutting them into blocks and socking in 25 mL of 95% ethanol in darkness overnight. The absorbances of the solution were measured at 470, 649, and 665 nm using an ultraviolet spectrophotometer (L5, Shanghai Yidian Analytical Instrument Co., Ltd.). Total chlorophyll, chlorophyll a, and chlorophyll b contents were then calculated based on established methods (Nie et al. 2017).

### Transmission electron microscope (TEM) observation

Fresh root tips and leaf sections from W4 and 9311 were cut for TEM analysis (Cai et al. 2011). The samples were initially fixed overnight in 2.5% pentanediol in 0.1 M phosphate buffer (pH 7.0), followed by fixation in 1% osmium acid. Subsequently, the samples underwent a gradient dehydration process using alcohol concentrations of 30%, 50%, 70%, 80%, 90%, 95%, and finally 100% for 1 h each, before being embedded in spur resin. The embedded samples were then sectioned using a slicer (LEICA EM UC7, Wetzlar, Germany), stained with uranyl acetate and alkaline lead citrate for 5–10 min each, and observed using a TEM (Hitachi Model H-7650, Tokyo, Japan).

### Extraction and determination of antioxidant activity

Malondialdehyde (MDA) content was assessed using the 2-thiobarbituric acid method. Fresh samples weighing 2.5 g were cut into pieces and placed in a pre-cooled mortar. Phosphate buffer (pH 7.8) was then added and the samples were ground to a homogenate in an ice bath. The resulting homogenates were centrifuged at 4 °C, 8000 rpm for 25 min. The supernatant was utilized for determining MDA content, as well as superoxide dismutase (SOD), peroxidase (POD), and catalase (CAT) activity (Wang et al. 2009).

### Subcellular component Cd extraction and Cd speciation

Fresh samples weighing 0.30 g were homogenized in 6 mL of a pre-cooled extraction solution containing 250 mM sucrose, 50 mM Tris–HCl (pH 7.5), and 1 mM dithioerythritol. The homogenate was then centrifuged at 4 °C, 3000 rpm for 15 min to obtain the cell wall fraction. The supernatant was further centrifuged at 4 °C, 15000 rpm for 40 min, yielding the soluble cytoplasmic fraction in the supernatant and the cell organelles fraction in the sediment. The extracted fractions were digested with 5 mL of HNO_3_ in a heating block, and the Cd concentration was subsequently determined using ICP-MS (ICAP RQ, Thermo Fisher Scientific Co. Ltd., Waltham, USA) (Zhao et al. 2015b).

Chemical forms of Cd within plants were extracted using a series of different extracts in a step-by-step process: (I) 80% ethanol was used to extract inorganic Cd, with priority given to nitrate/nitrite, chloride, and aminophenol Cd; (II) Deionized water was used to extract water-soluble Cd of organic acid and M(PO_4_)_2_; (III) 1 M NaCl was used to extract pectates and protein integrated Cd; (IV) 2% HAc was used to extract undissolved Cd phosphate, including CdHPO_4_ and Cd_3_(PO_4_)_2_; (V) 0.6 M HCl was used to extract Cd oxalic; (VI) residual forms (Wu et al. 2005). In summary, a 5 g fresh sample was extracted with a 20 mL solution at 30 °C for 20 h in an incubator, then centrifuged at 3500 rpm for 10 min. The supernatant was separated, and the sediments were used for the next species extraction. The supernatants were evaporated at 70 °C, digested with 5 mL HNO_3_, and the Cd concentration was determined by ICP-MS (ICAP RQ, Thermo Fisher Scientific Co. Ltd., Waltham, USA) (Xin and Huang 2014).

### Statistical analysis

The data was analyzed by DPS 2000 and Origin 2021, and shown as means ± standard deviation. The statistical significance detection is carried out by Tukey’s test.

## Supplementary Information

Supplementary Material 1: Fig. S1. Fe, Mn, Cu and Zn contents in rice seedling roots and shoots of W4 and 9311 in the presence of 10 μM CdCl_2_ for 14 d. Data were shown as means ± S.D., n = 3. * and ** indicate significant differences at *P* < 0.05 or *P* < 0.01 by Tukey’s test, respectively. Table S1. Effects of Cd treatment on chlorophyll content of different rice cultivars. Table S2. Effect of Cd treatment on root morphological parameters. Table S3. Effects of Cd treatment on photosynthetic characteristics of different rice cultivars.

Supplementary Material 2.

## Data Availability

The datasets generated during and/or analysed during the current study are available from the corresponding author on reasonable request.

## Abbreviations

Cd: Cadmium
OsHMA3: Heavy metal associated protein 3 in rice
JECFA: The Joint FAO/WHO Expert Committee on Food Additives
*P*_*n*_: Net photosynthetic rate
*G*_*s*_: Stomatal conductance
*T*_*r*_: Transpiration rate
TEM: Transmission electron microscope
ICP-MS: Inductively Coupled Plasma Mass Spectrometry
MDA: Malondialdehyde
SOD: Superoxide dismutase
POD: Peroxidase
CAT: Catalase
PCA: Principal Component Analysis
