# Supplementary material for: Distinct cadmium detoxicity response between rice cultivars carrying functional or loss-of-function OsHMA3 allele
Source: Stress Biol. 2026 Oct 15;6(1):70. doi: 10.1007/s44154-025-00222-z (PMC13672768; doi:10.1007/s44154-025-00222-z)
Supplement: Supplementary file 1 [file 44154_2025_222_MOESM1_ESM.docx]

**Distinct Cadmium detoxicity response between rice cultivars carrying functional or loss-of-function *OsHMA3* allele**

Fuqing Sui^1^, Jing Tian^1^, Ziyuan Yang^1^, Yanzheng Yang^1^, Chang Li^1^, Hongen Liu, Jiali Yan^2*^, and Peng Zhao^1*^

1. Key Laboratory of Soil Pollution Control and Remediation of Henan Province, College of Resources and Environment, Henan Agricultural University, Zhengzhou, China

2. College of Civil and Architecture Engineering, Chuzhou University, Chuzhou, 239000, China

* Correspondence: zhaopeng@henau.edu.cn or jialiyan092@163.com. No. 218, Pingan Rd., Jinshui Dist., Zhengzhou, China. 450002.

**Supplementary materials**

**Table S1** Effects of Cd treatment on chlorophyll content of different rice cultivars

| Treatment | Chlorophyll contents  (mg g-1 FW) | | | Carotenoid contents  (mg g-1 FW) |
| --- | --- | --- | --- | --- |
|  | a | b | a+b |  |
| W4-CK | 34.62±0.38a | 15.65±1.39a | 50.26±1.01a | 8.23±0.35a |
| 9311-CK | 34.67±0.36a | 9.14±0.3b | 43.84±2.23b | 8.88±0.29a |
| W4-Cd10 | 23.88±0.33b | 8.98±0.35b | 32.86±0.66c | 4.53±0.16b |
| 9311-Cd10 | 31.69±3.68a | 9.97±1.07b | 41.66±2.61b | 8.40±0.80a |

The rice seedlings were hydroponically treated with or without 10 μM Cd for 14 d. Data were shown as means ± S.D., n = 3. FW, Fresh weight. Different lower cases indicate significant differences at *P* < 0.05 by Tukey’s test.

**Table S2** Effect of Cd treatment on root morphological parameters

| Treatment | Root length (cm) | Root surface area (cm^2^) | Root diameter (mm) | Root volume (cm^3^) | Root tip number |
| --- | --- | --- | --- | --- | --- |
| W4-CK | 354.90±28.49a | 46.34±5.56b | 0.40±0.02bc | 354.90±28.49b | 1912±104.89a |
| 9311-CK | 362.50±12.70a | 56.85±4.60a | 0.47±0.02a | 469.93±65.49a | 2001.24±125.80a |
| W4-Cd10 | 238.54±30.87b | 26.26±3.96c | 0.35±0.03c | 238.54±30.87c | 1374.78±126.20b |
| 9311-Cd10 | 361.24±6.28a | 49.76±3.93ab | 0.44±0.03ab | 361.24±6.28b | 2031.70±100.75a |

The rice seedlings were hydroponically treated with or without 10 μM Cd for 14 d. Data were shown as means ± S.D., n = 3. Different lower cases indicate significant differences at *P* < 0.05 by Tukey’s test.

**Table S3** Effects of Cd treatment on photosynthetic characteristics of different rice cultivars

| Treatment | *P_n_*/(μmol·m^-2^·s^-1^) | *G_s_*/(mol·m^-2^·s^-1^) | *C_i_*/(μmol·mol^-1^) | *T_r_*/(mmol·m^-2^·s^-1^) |
| --- | --- | --- | --- | --- |
| W4-CK | 25.34±1.45b | 0.09±0.01a | 449.73±16.41a | 1.68±0.2a |
| 9311-CK | 35.00±2.69a | 0.12±0.02a | 470.08±1.78a | 1.97±0.24a |
| W4-Cd10 | 23.55±0.49b | 0.03±0.002b | 343.11±66.76b | 0.47±0.08c |
| 9311-Cd10 | 32.48±3.50a | 0.05±0.003ab | 467.54±32.93a | 0.91±0.05b |

The rice seedlings were hydroponically treated with or without 10 μM Cd for 14 d. Data were shown as means ± S.D., n = 3. Different lower cases indicate significant differences at *P* < 0.05 by Tukey’s test.

**Fig. S1** Fe, Mn, Cu, and Zn contents in rice seedling roots and shoots of W4 and 9311 in the presence of 10 μM Cd for 14 d. Data were shown as means ± S.D., n = 3. * and ** indicate significant differences at P < 0.05 or P < 0.01 by Tukey’s test, respectively.
